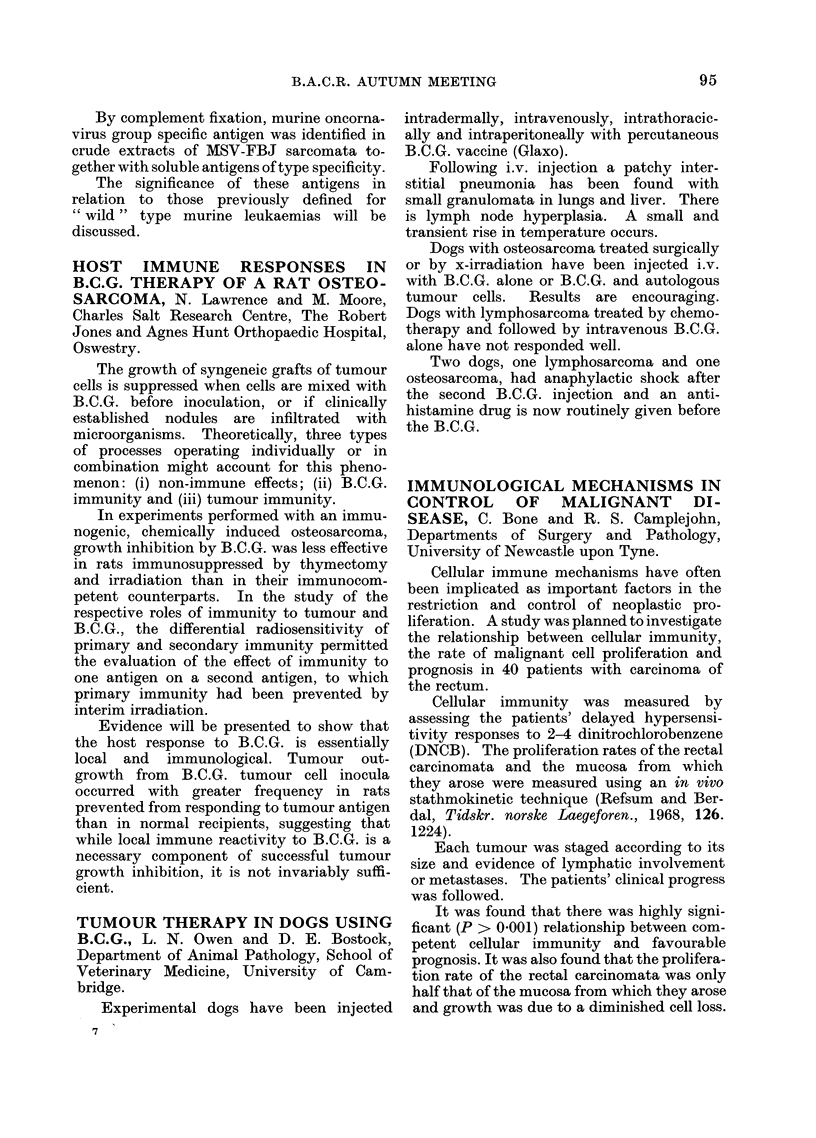# Proceedings: Host immune responses in B.C.G. therapy of a rat osteosarcoma.

**DOI:** 10.1038/bjc.1974.23

**Published:** 1974-01

**Authors:** N. Lawrence, M. Moore


**HOST IMMUNE RESPONSES IN B.C.G. THERAPY OF A RAT OSTEOSARCOMA,** N. Lawrence and M. Moore, Charles Salt Research Centre, The Robert Jones and Agnes Hunt Orthopaedic Hospital, Oswestry.

The growth of syngeneic grafts of tumour cells is suppressed when cells are mixed with B.C.G. before inoculation, or if clinically established nodules are infiltrated with microorganisms. Theoretically, three types of processes operating individually or in combination might account for this phenomenon: (i) non-immune effects; (ii) B.C.G. immunity and (iii) tumour immunity.

In experiments performed with an immunogenic, chemically induced osteosarcoma, growth inhibition by B.C.G. was less effective in rats immunosuppressed by thymectomy and irradiation than in their immunocompetent counterparts. In the study of the respective roles of immunity to tumour and B.C.G., the differential radiosensitivity of primary and secondary immunity permitted the evaluation of the effect of immunity to one antigen on a second antigen, to which primary immunity had been prevented by interim irradiation.

Evidence will be presented to show that the host response to B.C.G. is essentially local and immunological. Tumour outgrowth from B.C.G. tumour cell inocula occurred with greater frequency in rats prevented from responding to tumour antigen than in normal recipients, suggesting that while local immune reactivity to B.C.G. is a necessary component of successful tumour growth inhibition, it is not invariably sufficient.